# A case report of epiploic appendagitis as a mimic of acute cholecystitis

**DOI:** 10.1016/j.ijscr.2018.11.003

**Published:** 2018-11-13

**Authors:** Emily Chan, Alaa El-Banna

**Affiliations:** NHS Western Isles, Western Isles Hospital, Macaulay Road, Stonorway, HS12AF, United Kingdom

**Keywords:** Case report, Epiploic appendagitis, Unusual presentation, Diagnostic mimic

## Abstract

**Introduction:**

Epiploic appendagitis is a rare cause of acute abdominal pain. It's presentation often mimics other causes of acute abdominal pain. It is important for clinicians to be aware of an epiploic appendagitis since a delay in diagnosis can lead to unnecessary hospital stay, antibiotic usage and surgical intervention [[1], [2], [3], [4]]).

We present a case of epiploic appendagitis mimicking acute cholecystitis in a rural community hospital.

**Case report:**

A 54 Caucasian male self-presents to the emergency department on a Saturday with severe right upper quadrant pain. He was initially diagnosed as acute cholecystitis and managed with antibiotics. Due to limitations with out-of-hours radiology, an ultrasound (US) scan was performed two days after admission. This US showed no evidence of cholecystitis or gallstones. A computed tomography (CT) scan was subsequently performed which revealed a diagnosis of epiploic appendagitis. The patient was discharged with analgesia and anti-inflammatories.

**Discussion:**

Epiploic appendagitis can mimic common pathologies causing acute abdominal pain. Unlike its mimics, epiploic appendagitis is a self-limiting condition and its initial management remains conservative. It is diagnosed with imaging studies such as CT scans. It is important for clinicians to be aware of an epiploic appendagitis as a cause for abdominal pain since a delay in diagnosis can lead to unnecessary hospital stay, antibiotic usage and surgical intervention [[1], [2], [3], [4]].

**Conclusion:**

A diagnosis of epiploic appendagitis should remain on the list of differential diagnosis for acute abdominal pain. A prompt diagnosis of epiploic appendagitis avoids unnecessary surgical intervention and antibiotic usage.

## Highlights

• Epiploic appendagitis can mimic acute cholecystitis and other common causes of abdominal pain.
• Epiploic appendagitis should be included in the differential of acute abdominal pain.
• Epiploic appendagitis is initially managed conservatively with analgesia and anti-inflammatories.

## 1 Introduction

Epiploic appendagitis, also known as appendicitis epiploica, hemorrhagic epiploitis, epiplopericolitis or appendagitis, is a rare cause of acute abdominal pain [5,6]. Epiploic appendagitis is the ischemic infarction of the epiploic appendages caused by torsion or spontaneous thrombus of the central draining vein. Its presentation often mimics other causes of acute abdominal pain such as appendicitis and diverticulitis [7]. Unlike its mimics, epiploic appendagitis is a self-limiting condition and initial management remains conservative. It is important for clinicians to be aware of epiploic appendagitis as a cause for abdominal pain since a delay in diagnosis can lead to prolonged hospital stay, antibiotic usage and surgical intervention [[1], [2], [3], [4]].

We present a case of epiploic appendagitis mimicking acute cholecystitis in a rural community hospital.

This case report has been reported in line with the SCARE criteria [8].

## 2 Presenting case

A 54 Caucasian male of normal body habitus self-presents to the emergency department of a rural community hospital with severe right upper quadrant pain (RUQ). Two days prior, he described a mild dull ache in his abdomen. On the day of admission, he developed a sudden onset severe sharp RUQ pain which was sore on inspiration, coughing and walking. No nausea, vomiting, fever or change in bowel habits.

Nil past medical or surgical history.

On examination, he had a soft abdomen with exquisite tenderness localized to the RUQ.

A chest X-ray showed lung field markings throughout with no consolidation, effusion, collapse or air under the diaphragm. Abdominal X-ray showed extensive fecal loading on the right colon with small segment of small bowel dilated loops. Laboratory findings included a white cell count of 8.0 × 10^9/I, C-reactive protein 30 mg/L with normal liver function tests and amylase.

With a presentation of acute RUQ pain and mildly raised inflammatory markers, he was initially diagnosed as acute cholecystitis and was managed with analgesia and antibiotics (intravenous amoxicillin, metronidazole and gentamicin) as per hospital guidelines. Throughout his admission, his vital signs remained normal and his pain improved with simple analgesia such as paracetamol.

**Fig. 1:**
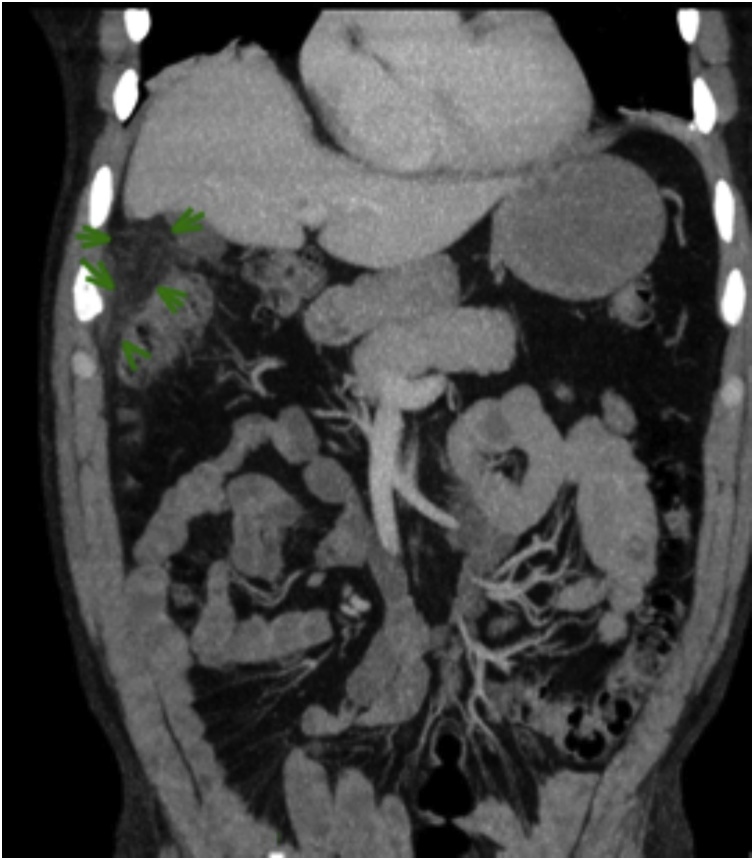
CT abdomen showing a fat density structure, approximately 61 × 25 × 25 mm, abutting the fundus of the gallbladder and the adjacent liver and is in keeping with epiploic appendagitis).

An abdominal ultrasound (US) was performed two days after admission due to the limitations of out-of-hours radiology over the weekend. This ultrasound report showed a contracted gallbladder, no gallstones and no evidence of cholecystitis. In view of his initial examination of a localized and exquisite abdominal tenderness, a computed tomography (CT) scan was arranged to exclude other differentials such as high retrocecal appendix, diverticulitis or perforated viscus. The CT scan showed a gallbladder of unremarkable appearance with no intra or extrahepatic biliary dilatation. There is a fat density structure, approximately 61 × 25 × 25 mm, abutting the fundus of the gallbladder and the adjacent liver. This structure is inseparable from the hepatic flexure appearances is in keeping with acute epiploic appendagitis arising from hepatic flexure, abutting the non-inflamed gallbladder (see Fig. 1). A diagnosis of epiploic appendagitis was made on CT scan.

His antibiotic therapy was stopped, and he was discharged with advice and simple analgesia (paracetamol). No further follow-up organized.

## 3 Discussion

Epiploic appendagitis, first described in 1956 by Dockerty et al. [6], is the ischemic infarction of epiploic appendage secondary to torsion or spontaneous thrombosis of the central draining vein [9]. It is an uncommon cause for abdominal pain. It’s frequency is estimated to be 1.3% with an incidence of 8.8 cases/million/year [10]. A diagnosis of epiploic appendagitis is usually an incidental finding and is found when investigating for other causes of acute abdominal pain [7,9]. Epiploic appendagitis is rare and should not be considered as a top differential diagnosis for abdominal pain; however knowledge of epiploic appendagitis is important amongst a multitude of specialties as it can mimic common causes of acute abdominal pain [9]. A prompt diagnosis of epiploic appendagitis avoids unnecessary surgical intervention and antibiotic usage. Appendagitis usually presents as an acute lower abdominal pain and can mimic common abdominal pathologies such as appendicitis or diverticulitis [7]. It rarely presents as a cause of upper abdominal pain.

Epiploic appendages are outpouching of fat which are found on the external surface of the colon [9]. There exist roughly 50–100 epiploic appendages in the colon with the more common and larger appendages existing in the transverse and sigmoid colon [11]. Epiploic appendagitis occurs commonly between 20–50 years of age and risk factors include being male, obesity and strenuous exercise [9]. Men have a 4× greater predisposition than woman of developing epiploic appendagitis [12].

Epiploic appendagitis can be primary or secondary. Primary epiploic appendagitis is caused by torsion when the appendage is long and large. Secondary epiploic appendagitis is caused by adjacent inflammatory pathologies such as diverticulitis or appendicitis [14]. Presentation is typically with acute or subacute abdominal pain. It has been reported in 2–7% of those initially diagnosed with acute diverticulitis and 0.3–1% in those diagnosed with acute appendicitis [13]. The majority (60–80%) present in the left lower quadrant, but can also occur in the right lower quadrant [7]. The majority occur in the rectosigmoid (57%) or ileocecum (26%) which accounts for their common mimic of lower abdominal pain. Rarer occurrences occur in the ascending (9%), transverse (6%) and descending colon (2%) [13]. The pain is often characterized as dull, localized and without radiation [12]. Rarely associated with other symptoms such as vomiting, bloating, diarrhea, and early satiety [11]. Examination typically shows localized abdominal pain with normal or mildly elevated inflammatory markers such as white cell count and C-reactive protein. Diagnosis is often an incidental finding when investigating for other commoner causes of acute abdominal pain. Diagnosis is made primarily by CT scan, however US and magnetic resonance imaging (MRI) can be used if CT isn't available or is equivocal [15]. Normally, epiploic appendages are not seen on CT scan unless surrounded by intra-peritoneal fluid or inflammation [9]. With epiploic appendagitis, CT scan usually shows an oval shaped fat density paracolic mass with thickened peritoneal lining and periappendageal fat stranding [13]. US may show an inflamed appendage as a non-compressible solid hyperechoic mass connected to the adjacent colon [11]. MRI scan is rarely used for diagnosis but will show a focal lesion that shares a similar intensity to fat. T1 weighted images show an enhancing rim around the oval fatty lesion [2].

A diagnosis of epiploic appendagitis should be considered in a patient presenting with acute localized peritoneal non-migratory pain without anorexia, fever, or sepsis. These patients may benefit from an early CT scan to diagnose other common causes of acute abdominal pain since epiploic appendagitis is rarer. An early diagnosis of epiploic appendagitis may save the patient from unnecessary antibiotics and surgery [1]. Management is mostly conservative with anti-inflammatory medications and simple analgesia. Epiploic appendagitis is a self-limiting condition which resolves within 3–14 days without the need for antibiotics and surgery [7]. Surgery is warranted in those who fail with medical management or who exhibit complications such as obstruction, intussusception and abscess formation [14].

In our case, epiploic appendagitis was not one of the main differential diagnosis considered. Our decision to admit and treat with antibiotics and analgesia was based on the clinical judgement that this patient had acute cholecystitis. The delay in obtaining immediate investigations is explained by the limited resources during the out-of-hour period, and due to the fact that the patient was stable and did not warrant urgent CT scan out-of-hours. After careful investigation leading to a diagnosis of epiploic appendagitis, the patient was discharged 1 to 2 days after diagnosis. The decision for CT scanning prevented the need for unnecessary surgery and treatment in our patient.

Although many cases of epiploic appendagitis exist, not many initially present with RUQ pain mimicking acute cholecystitis. There should be an awareness of the existence of epiploic appendagitis presenting as RUQ. A diagnosis of epiploic appendagitis should be included within a list of differential diagnosis for RUQ abdominal pain after common causes have been excluded.

## 4 Conclusion

Unlike its mimics, epiploic appendagitis is a self-limiting condition and its initial management remains conservative. It is important for clinicians to be aware of an epiploic appendagitis as a cause for abdominal pain since a delay in diagnosis can lead to unnecessary hospital stay, antibiotic usage and surgical intervention [1,2].

## Conflicts of interest

Nil.

## Sources of funding

Nil.

## Ethical approval

N/A.

## Consent

Written consent obtained from the patient.

## Author contributions

Emily Chan Conceptualization, Validation, Investigation, Writing-original draft, Visualization. Alaa El-Banna Writing- review & editing, Supervision.

## Registration of research studies

N/A.

## Guarantor

Emily Chan.

## Peer review and provenance

Not commissioned, externally peer reviewed.
